# Current Status and Future of Artificial Intelligence in Medicine

**DOI:** 10.7759/cureus.77561

**Published:** 2025-01-16

**Authors:** Omar Basubrin

**Affiliations:** 1 Department of Medicine, Umm Al-Qura University, Makkah, SAU

**Keywords:** artificial intelligence, deep learning, diagnostics, healthcare, machine learning, medicine, natural language processing, treatment

## Abstract

Artificial intelligence (AI) has rapidly emerged as a transformative force in medicine, revolutionizing various aspects of healthcare from diagnostics and treatment to public health and patient care. This narrative review synthesizes evidence from diverse study designs, exploring the current and future applications of AI in medicine. We highlight AI's role in improving diagnostic accuracy, optimizing treatment strategies, and enhancing patient care through personalized interventions and remote monitoring, drawing upon recent advancements and landmark studies. Emerging trends such as explainable AI and federated learning are also examined. While acknowledging the tremendous potential of AI in medicine, the review also addresses the barriers and ethical challenges that need to be overcome, including concerns about algorithmic bias, transparency, over-reliance, and the potential impact on the healthcare workforce. We emphasize the importance of establishing regulatory guidelines, fostering collaboration between clinicians and AI developers, and ensuring ongoing education for healthcare professionals. Despite these challenges, the future of AI in medicine holds immense promise, with the potential to significantly improve patient outcomes, transform healthcare delivery, and address healthcare disparities.

## Introduction and background

Artificial Intelligence (AI) is at the forefront of the Fourth Industrial Revolution, an era characterized by rapid technological advancements and the fusion of physical, digital, and biological worlds [1]. This represents a paradigm shift in how we interact with and utilize technology. In the context of medicine, AI encompasses a broad spectrum of computational tools and techniques that enable machines to perform tasks typically requiring human intelligence, such as pattern recognition, decision-making, learning, and natural language processing (NLP) [1]. AI systems employ sophisticated algorithms to identify trends and anomalies in large datasets, enabling them to generate outputs that may resemble informed decisions. They can also be designed to adjust their parameters based on new data, and through techniques like NLP, they can analyze and generate human language in a way that simulates understanding [1].

AI in medicine manifests in various subtypes, each with unique functionalities. Machine learning, a type of AI that allows algorithms to learn from data and improve their performance without being explicitly programmed, is particularly useful for identifying patterns and trends in medical data that may not be apparent to human experts. Deep learning, a subset of machine learning that uses artificial neural networks with multiple layers, is well-suited for analyzing complex patterns in data, such as images, natural language, and genomic sequences. NLP enables machines to understand, interpret, and generate human language, facilitating tasks like extracting information from medical records, summarizing patient histories, and generating personalized treatment plans. Computer vision empowers machines to interpret and understand visual information from the world, such as images and videos, which is essential for medical image analysis, detecting abnormalities in X-rays or MRIs, and assisting in surgical procedures [1].

While the capabilities of AI systems may sometimes evoke comparisons to human intelligence, it is important to recognize that they operate fundamentally differently. AI's remarkable achievements stem from its ability to process vast amounts of data and identify patterns through complex algorithms, rather than possessing true understanding or independent thought in the human sense. Throughout this review, we will use terms like "decision-making" or "understanding" to describe AI's outputs, but it is crucial to remember that these are metaphorical representations of its computational processes, not reflections of genuine human-like cognition.

The past decade has witnessed an exponential rise in AI's application within medical science, making it one of the most heavily funded sectors globally [2]. AI has revolutionized healthcare, impacting patient triage, diagnostics, personalized treatment plans, and monitoring. This integration has significantly improved outcomes for both clinicians and patients.

This narrative review encompasses a broad range of literature on AI in medicine, including peer-reviewed articles, conference proceedings, reputable websites, and grey literature. The search focused on key themes such as AI applications in diagnostics, treatment, public health, and patient care, with particular emphasis on recent advancements and landmark studies. While striving to encompass diverse perspectives and minimize potential bias, the selection process was guided by the relevance and significance of the findings to the overall narrative, prioritizing studies that offered insights into the current status and future prospects of AI in medicine.

## Review

AI technologies in healthcare

Healthcare leverages a diverse array of AI technologies. Machine learning, a foundational AI form, employs algorithms that emulate human learning and is currently utilized for predicting treatment protocols. Common machine learning algorithms used in healthcare include decision trees, support vector machines, and random forests. These algorithms can be trained on large datasets to identify patterns and make predictions about patient outcomes or treatment responses [3]. Subsets of machine learning, such as neural networks and deep learning, contribute to deciphering a patient's genetic makeup and identifying clinically relevant symptoms, respectively. Deep learning architectures like convolutional neural networks (CNNs) have shown remarkable success in medical image analysis, enabling accurate detection of tumors, fractures, and other abnormalities [4].

NLP, another AI application, aids in generating reports and transcribing patient-doctor interactions. NLP techniques like named entity recognition and sentiment analysis can extract valuable information from unstructured clinical notes and social media data, providing insights into patient experiences and disease trends [5]. Additionally, AI-powered surgical robots enable precise, minimally invasive procedures, while robotic process automation streamlines patient records and billing [6].

Beyond these applications, AI is also being used in areas like drug discovery, clinical trial design, and precision medicine, demonstrating its wide-ranging potential in healthcare. For instance, AI-powered platforms are being used to analyze vast amounts of genomic and clinical data to identify potential drug targets and predict drug efficacy, potentially accelerating the drug discovery process [7].

AI's expanding role in diagnostics: benefits and challenges

Timely and accurate disease diagnosis remains a significant challenge for physicians. AI's role in diagnostics, although still evolving, shows great promise. Numerous global studies have demonstrated its efficacy as comparable, if not superior, to human capabilities in specific tasks.

AI has shown remarkable potential in various medical domains. For example, in a Turkish study comparing cardiologists, emergency physicians, and AI, AI outperformed both human experts in interpreting ECG abnormalities [8]. Additionally, AI has demonstrated superior sensitivity and earlier detection of breast cancer with definitive mass compared to radiologists, as evidenced by a South Korean study [9]. Furthermore, AI has proven effective in diagnosing skin cancers, with biopsy results validating its accuracy [10]. Beyond cancer, AI aids in diagnosing acute conditions like tuberculosis, pneumonia, and acute appendicitis [11-13].

AI expedites the identification of causative organisms, enabling targeted antibiotic prescriptions. AI-powered genomic sequencing has identified infectious agents like COVID-19 and malaria. Its cost-effectiveness and time efficiency make it a valuable asset in clinical laboratories [14]. Furthermore, AI excels in rapidly interpreting medical imaging like CT scans, MRIs, and bone scans, potentially improving patient survival through early diagnosis and timely treatment initiation.

AI also shows promise in enabling personalized medicine by predicting disease risk and tailoring treatment plans based on individual patient characteristics. Recent studies have shown that AI can accurately predict the risk of developing conditions like cardiovascular disease and diabetes, allowing for early interventions and preventive measures [15].

Limitations and Challenges to AI in Diagnostics

While AI holds tremendous potential in diagnostics, it is essential to acknowledge its limitations and potential pitfalls. A key concern is the potential for algorithmic bias, which can lead to disparities in diagnosis and treatment recommendations, particularly for underrepresented populations. Ensuring fairness and equity in AI-driven diagnostics is a critical challenge that necessitates careful attention to data collection, algorithm development, and ongoing monitoring. The use of diverse and representative datasets, along with techniques like fairness constraints and adversarial debiasing, can help mitigate algorithmic bias and ensure equitable outcomes for all patients [16]. The potential for algorithmic bias, particularly in underrepresented populations, remains a significant concern and a subject of ongoing research and debate.

Another challenge lies in the "black box" nature of some AI models. The complexity of these models can make it difficult for clinicians to understand the underlying reasoning behind AI-generated diagnoses. This lack of transparency can undermine trust and hinder the acceptance of AI in clinical practice. Explainable AI, a growing field of research, aims to address this challenge by developing methods to make AI models more interpretable and understandable to humans. Techniques like attention mechanisms, feature importance visualization, and rule-based explanations can provide insights into the decision-making process of AI models, fostering trust and facilitating clinical adoption [17].

Furthermore, the risk of over-reliance on AI, leading to the deskilling of human clinicians, is another concern that needs to be addressed. It is imperative to ensure that AI serves as a supportive tool for clinicians rather than a replacement, promoting a collaborative approach to diagnosis. The current legal and regulatory landscape offers little clarity on liability in cases where AI contributes to diagnostic or therapeutic errors, underscoring the need for careful collaboration and shared responsibility between human clinicians and AI systems. The integration of AI into clinical workflows should be designed to enhance the capabilities of clinicians, allowing them to focus on complex cases and patient interaction while AI handles routine tasks and provides valuable insights.

AI in the emergency department: enhancing efficiency and patient outcomes

AI holds particular promise in the emergency department, where it can enhance efficiency and patient outcomes amid increasing patient volumes. AI has shown benefits in diagnostics, aiding in the identification of conditions like pneumothorax, stroke, acute coronary syndrome, sepsis, and traumatic brain injuries [18]. AI-driven diagnoses are often faster and more cost-effective than those made by humans, potentially leading to improved patient outcomes in time-sensitive situations. For example, a recent study demonstrated that an AI model could accurately detect intracranial hemorrhage on CT scans with sensitivity and specificity comparable to that of experienced radiologists, potentially expediting diagnosis and treatment in stroke patients [19].

In addition to diagnostics, AI contributes to effective triage, particularly for trauma victims, by predicting patient outcomes and determining the urgency of care and the need for admission [20]. This can help prioritize patients who require immediate attention, potentially saving lives. AI can also assist in optimizing resource allocation and streamlining workflows in the emergency department, leading to improved overall efficiency and patient satisfaction. AI-powered tools can predict patient flow, optimize staffing levels, and automate administrative tasks, freeing up clinicians to focus on patient care [21]. For instance, AI-based triage systems can analyze patient data and symptoms to assign acuity levels and prioritize patients based on their need for immediate medical attention, improving patient flow and reducing waiting times in the ED.

The Role of Human Clinicians in AI Emergency Departments

Despite these promising applications, it is crucial to recognize that AI is not intended to replace human clinicians in the emergency department. Instead, it should be viewed as a powerful tool that can augment their decision-making capabilities and improve efficiency. The final diagnosis and treatment decisions should always rest with the clinician, who can take into account the nuances of individual cases and the patient's overall clinical context. The human touch, empathy, and the ability to build rapport with patients remain irreplaceable aspects of healthcare that AI cannot replicate. It is important to emphasize that AI should be used to support and enhance the work of clinicians, not to replace them.

AI in treatment strategies: personalized medicine and beyond

Modern healthcare emphasizes personalized medicine, tailoring treatment to individual patient characteristics. AI has already achieved remarkable success in robotic and laparoscopic surgeries, enabling precise and minimally invasive procedures. AI-powered surgical robots can assist surgeons in performing complex procedures with greater accuracy and dexterity, reducing complications and improving patient outcomes [22]. Furthermore, AI assists in customizing treatments based on genomics, risk factors, lifestyle, and environment [23]. Advanced algorithms predict drug responses, such as those of antidepressants, allowing for dose optimization [24].

In a study of 175 cancer patients, AI-based algorithms were employed to assess responses to standard chemotherapeutic drugs. These algorithms proved particularly valuable in identifying alternative treatment options when initial therapies failed, demonstrating AI's potential to optimize cancer treatment plans [25]. AI also optimizes drug dosages and maintains therapeutic blood levels. The CURATE.AI platform, tested in a feasibility trial on 10 patients with advanced solid tumors, delivered capecitabine doses tailored to individual comorbidities and organ dysfunctions. Preliminary findings suggest its potential for predicting patient outcomes and improving drug responses [26].

By considering a patient's individual genetic makeup, AI can help determine therapeutic and toxic drug ranges, optimize treatment, predict drug interactions, and identify potential adverse events [27]. This leads to optimal drug concentrations and minimizes toxicity. AI is also being explored in the development of new drugs and therapies, potentially accelerating the drug discovery process and bringing novel treatments to patients faster. For example, AI-powered platforms are being used to screen vast libraries of chemical compounds and predict their potential therapeutic effects, significantly reducing the time and cost associated with traditional drug discovery methods [28]. Additionally, AI can analyze large clinical trial datasets to identify patterns and predict treatment responses, leading to more efficient and targeted clinical trials [29].

Navigating the Complexities in Treatment

While AI holds immense potential in treatment strategies, several complexities and challenges need to be addressed. One significant hurdle is the need for large, high-quality datasets to develop robust and generalizable AI models. Obtaining such datasets can be challenging due to privacy concerns, data fragmentation across different healthcare systems, and the inherent variability in patient populations and disease presentations. Collaborative efforts between healthcare institutions, data-sharing initiatives, and the development of standardized data collection protocols can help overcome these challenges and facilitate the development of effective AI models for treatment optimization [30].

Another complexity arises from the dynamic and multifaceted nature of human biology and disease processes. AI algorithms need to account for this complexity and adapt to evolving patient conditions and treatment responses. While AI excels at identifying patterns in large datasets, it may struggle to capture the nuances of individual patient circumstances, including epigenetic factors and the complex interplay of internal and external influences on health.

The use of longitudinal data, continuous learning algorithms, and real-time monitoring can enable AI models to adapt to changing patient conditions and provide personalized treatment recommendations throughout the course of the disease [31].

Additionally, integrating AI into existing clinical workflows can be challenging, requiring careful consideration of user interfaces, interoperability with electronic health record systems, and the potential impact on clinician workload and decision-making processes. User-centered design principles, seamless integration with existing systems, and comprehensive training programs for clinicians can facilitate the smooth adoption of AI in clinical practice [32].

AI and Human Collaboration in Treatment

The successful implementation of AI in treatment strategies hinges on effective collaboration between AI and human clinicians. AI can provide valuable insights and recommendations based on its analysis of vast amounts of data, but the ultimate responsibility for treatment decisions should remain with the clinician [33]. A synergistic approach, where AI complements human expertise, is crucial for achieving optimal patient outcomes. Clinicians need to be equipped with the knowledge and skills to critically evaluate AI-generated recommendations and integrate them into their clinical decision-making process. Transparent and explainable AI models, along with continuous education and training for clinicians, can foster trust and facilitate effective collaboration between humans and AI in the treatment process. This collaborative model ensures that the human touch and clinical judgment remain central to patient care, while AI provides valuable support and insights.

AI in public health

Public health management relies on data analysis and predictive modeling to enhance population health outcomes. By examining demographics, medical histories, lifestyle factors, and socioeconomic data, AI can identify at-risk populations and develop targeted interventions [34]. AI-based models analyze vast datasets, revealing patterns that inform specific interventions. Predictive models not only forecast disease burdens but also promote public awareness. Sehaa, an analytical tool used in Saudi Arabia, exemplifies this by identifying disease prevalence variations across cities [35]. AI's ability to determine disease burden, prevalence, and outcomes supports the implementation of nationwide health strategies. The use of AI in public health has the potential to improve disease surveillance, outbreak prediction, and resource allocation, leading to more effective and efficient public health interventions [36]. AI can also play a crucial role in analyzing social media data and other non-traditional sources of information to identify emerging health trends and potential outbreaks, enabling early interventions and proactive public health measures [37].

AI and patient care

The ever-growing demands of medicine and healthcare necessitate innovative solutions. AI has empowered healthcare providers through applications that offer medical advice based on symptoms, medication reminders, and vital sign monitoring. The NHS utilizes one such application for symptom-based triage, reducing unnecessary emergency room visits [38].

AI also plays a crucial role in mental health, with AI-driven tools delivering cognitive behavioral therapy (CBT) [39] and aiding in the treatment of anxiety, depression, and substance abuse. AI platforms enhance patient knowledge about their conditions, promoting smoking cessation and healthy lifestyles. Apps like ChatGPT and PROSCA have improved patient understanding of diabetes and prostate cancer, respectively [40,41]. These applications not only empower patients but also alleviate physician burden, potentially mitigating burnout [42].

Moreover, AI-powered chatbots and virtual assistants can provide 24/7 support to patients, answering their questions, providing medication reminders, and offering emotional support, all while complementing the essential role of human healthcare providers. This improves patient engagement and adherence to treatment plans [43]. AI can also facilitate remote patient monitoring, allowing clinicians to track patients' vital signs and symptoms in real time, enabling early detection of complications and timely interventions [44].

Regulatory landscape and standards

The rapid advancement of AI in medicine necessitates the establishment of clear regulatory guidelines and standards. These frameworks should ensure the safety, efficacy, and ethical use of AI technologies in healthcare settings. Regulatory bodies need to address issues such as data privacy, algorithmic transparency, and accountability for AI-driven decisions. International collaboration is crucial to harmonize regulatory approaches and facilitate the responsible development and deployment of AI in medicine. Initiatives like the FDA's Digital Health Software Precertification Program and the European Union's Medical Device Regulation are steps in the right direction, but continuous adaptation and refinement of regulatory frameworks are needed to keep pace with the rapid evolution of AI in healthcare [45]. It is crucial to strike a balance between encouraging innovation and ensuring patient safety and data protection.

Collaboration and education

Successful integration of AI in medicine requires close collaboration between clinicians, researchers, and AI developers. Clinicians possess valuable domain expertise, while researchers can contribute to the development and validation of AI algorithms. AI developers can then translate these algorithms into user-friendly and clinically relevant tools.

Additionally, ongoing education and training for healthcare professionals are essential to ensure they can effectively utilize and interpret AI-generated insights, fostering a culture of lifelong learning and adaptation to technological advancements. The development of interdisciplinary training programs, workshops, and conferences can facilitate knowledge exchange and collaboration between different stakeholders, promoting the responsible and effective use of AI in medicine [33]. Medical curricula should also be updated to include AI literacy and training, preparing future healthcare professionals for the AI-driven healthcare landscape.

Future prospects of AI in medicine

AI's impact on healthcare is already significant, but its potential for future advancements remains vast. Researchers are actively exploring sophisticated methods for patient data collection, advanced robotic surgeries, drug discovery, and virtual nursing assistants [46].

One particularly promising avenue is addressing healthcare disparities and improving access to care. AI-powered telemedicine and decision support tools can bring specialized expertise to underserved and remote areas, mitigating geographical barriers to quality healthcare [47].

Furthermore, AI's integration with emerging technologies like wearable devices, the Internet of Things (IoT), and blockchain holds immense potential. Continuous patient monitoring, real-time data collection, and secure data sharing can revolutionize healthcare delivery and empower both patients and clinicians [48]. For instance, AI-enabled wearables could allow for early detection of health deterioration, facilitating timely interventions [49], while blockchain could ensure the integrity and privacy of patient data across healthcare networks [50].

However, these advancements must be coupled with efforts to bridge the digital divide, ensuring that the benefits of AI are accessible to all, regardless of socioeconomic status or geographical location.

In essence, AI's future in medicine lies in its ability to augment human capabilities, improve access to care, and personalize treatment, ultimately leading to more efficient, effective, and equitable healthcare systems.

Barriers to AI in medicine

While AI has made significant strides in healthcare, several barriers impede its widespread implementation.

Data and Technological Barriers

These include the lack of high-quality, diverse medical data, potential data breaches, and challenges related to AI's performance with limited datasets [51]. Additionally, the "black-box" nature of many AI models can hinder trust and acceptance among healthcare professionals due to a lack of transparency in their decision-making processes.

Ethical and Social Barriers

The inability of AI to fully replicate human empathy and the potential for algorithmic bias raise ethical concerns [51]. Furthermore, apprehension about job displacement among healthcare professionals, along with patient concerns about data privacy and the dehumanization of care, pose additional social challenges.

Implementation and Workforce Barriers

The integration of AI into existing clinical workflows, the need for comprehensive training programs for healthcare staff, and the challenge of establishing clear lines of accountability in AI-assisted decision-making all contribute to implementation hurdles.

Addressing these barriers necessitates a multi-pronged approach. Investments in data infrastructure and the development of transparent, explainable AI models are crucial. Comprehensive training programs for healthcare professionals can foster trust and facilitate effective AI utilization. Moreover, clear regulatory guidelines are needed to ensure patient safety, data privacy, and algorithmic fairness. Importantly, fostering collaboration between technical and healthcare teams is essential for designing AI applications that genuinely benefit the healthcare industry. Encouraging patient interaction with AI and incorporating AI education into undergraduate medical curricula can further facilitate its adoption [52].

Ethical aspects of AI in medicine

The ethical implications of AI in medicine demand thorough consideration. While acts like the Health Insurance Portability and Accountability Act (HIPAA) and the General Data Protection Regulation (GDPR) address data privacy, the use of AI raises additional concerns [53,54]. These concerns include ongoing debates about the potential for algorithmic bias to perpetuate existing healthcare disparities, the risk of over-reliance on AI leading to the deskilling of healthcare professionals, and the challenge of ensuring transparency and explainability in AI-driven decision-making. Questions of accountability arise when AI contributes to medical decisions, potentially blurring the lines of responsibility between human clinicians and AI systems, a topic of intense debate in the medical community. Informed consent becomes complex when patients interact with AI-driven tools, requiring clear communication about the role of AI and its limitations, an area where ethical guidelines are still evolving.

Furthermore, as AI systems become more sophisticated, concerns about job displacement for healthcare professionals emerge, sparking discussions about the future of the healthcare workforce and the need for upskilling and retraining. While AI may automate certain tasks, it is also likely to create new roles and opportunities, necessitating a proactive approach to workforce development and training.

Lastly, the inherent limitations of AI in replicating human empathy and compassion raise concerns about its impact on the patient-clinician relationship [55]. Striking the right balance between technological advancement and preserving the human touch in healthcare remains a crucial ethical consideration. The development and implementation of AI in medicine should be guided by ethical principles that prioritize patient well-being, autonomy, and justice, ensuring that AI serves as a tool to enhance human capabilities rather than replace them. It is also important to consider the potential for AI to be used for malicious purposes, such as in the development of autonomous weapons or the spread of misinformation, and to implement safeguards to prevent such misuse [56].

## Conclusions

AI has undeniably ushered in a new era in medicine, with remarkable advancements already evident in diagnostics, treatment, public health, and patient care. The potential benefits of AI in revolutionizing healthcare delivery and improving patient outcomes are immense. However, the successful and responsible integration of AI in medicine hinges on addressing the associated challenges. These challenges encompass not only technical hurdles like data quality and algorithmic transparency but also ethical considerations such as algorithmic bias, the potential for the deskilling of healthcare professionals, and the need to preserve the human touch in patient care.

A collaborative approach involving clinicians, researchers, and AI developers is crucial to ensure that AI solutions are clinically relevant, technically robust, and ethically sound. Continued education and training for healthcare professionals are paramount to empower them to effectively utilize and interpret AI-generated insights, fostering a culture of lifelong learning and adaptation in the face of rapid technological advancement. The establishment of clear regulatory frameworks and the prioritization of ethical considerations are essential to ensure the safe, equitable, and responsible use of AI in healthcare. The future of AI in medicine is undeniably promising. By embracing collaboration, education, and ethical principles, we can harness the full potential of AI to transform healthcare delivery, improve patient outcomes, address healthcare disparities, and ultimately revolutionize the way we diagnose, treat, and prevent diseases. The path forward lies in a synergistic alliance between human expertise and AI capabilities, where technology serves as a powerful tool to augment and enhance, rather than replace, the human touch that remains at the core of patient care.
